# Abstract of Late Views on Some Points in the Physiology of Reproduction

**Published:** 1846-08

**Authors:** 


### PART V.—ABSTRACTS.

### ARTICLE XVIII.

### ABSTRACT OF LATE VIEWS ON SOME POINTS IN THE PHYSIOLOGY OF REPRODUCTION.

*The analogy of Menstruation with the rut or heat of Animals.*—Mr. Girdwood has, in the Lancet for December, 1844, set forth the following facts, established by observations on the dog, rabbits, cow and mare, viz: that the catamenial discharge appears in the lower animals as well as in the human female. The secretion is characterized also in them by a periodicity peculiar to each genus. It is, at least, in the higher order of mamals equally sanguineous. It attends the phenomena of rut or heat, and is microscopically and chemically more or less closely allied to that of the human female, being more similar the nearer we approach man in the scale of existence. It appears to be an increased periodic flow of the usual mucous secretions, with the addition, in the higher genera of animals, of more or less blood, which is, from its diffusion, deprived of its usual amount of coagulation. These correspond with results of the inquiries of Bischoff, Raciborski, and others.

In woman, and in female animals which exhibit the periodical discharge, it indicates the maturation of an ovum, its being on the point of discharge, and that the aptitude for conception is greatest at this period. In the human female the minute size of the ovum, and the rarity of opportunities of examining the bodies of those that have died during menstruation, render it difficult to prove this by the detection of ova; yet enough has been discovered to make the correctness of these views pretty certain.

With regard to animals, it has been proved by the experiments of Bischoff and Raciborski, that these are facts. Bischoff killed a lamb in rut for the first time, and which had never copulated, and found a Graafian vesicle ruptured in the right ovary, and an ovum in the fallopian tube of same side. He extirpated the left ovary and fallopian tube, (closing the wound by sutine) of a bitch, which was the second day in heat, and apparently desirous of receiving the male, but which was prevented, and found four Graafian vesicles in the ovary extremely turgid, but none had burst. After five days the bitch was killed; four large corpora lutea were found in the right ovary, and in the corresponding fallopian tube, four ova.

Besides this he has shown that the converse may take place. On an examination of the ovaries at as long a period as eighteen hours after coition, no ova had escaped, although spermatoza had reached the ovaries.—*Rankin's Abstract*, part 1.

In relation to menstruation, Dr. Carpenter says: "This flux of altered blood from the lining membrane of the uterus, is not confined to the human female, as was formerly supposed, but occurs in most of the lower mammalia, in the state of heat, or periodical aptitude for procreation, at which time the ovarium contains ova ready for impregnation."—*Elements of Physiology*, p. 433.

It has been urged against the identity of menstruation and the rut of animals that women are averse to sexual intercourse during the flow of the menses, whilst in animals, the male is only admitted during the period of rutting. This is answered by Bischoff and Girdwood, by considering the disinclination as the effect of habit and natural delicacy, rather than the evidence of any real disrelish, and that it is most probable that in human females, as well as in those of animals, the desire is greatest at this period, especially towards the decline of the discharge. It has been observed that bitches are languid and refuse the male during the first few days of heat, but after this they readily admit him. And this is considered analogous to the ailment of the human female in the early part of each menstrual period.—*Rankin's Abstract*, part 1.

*Spermatoza.*—It is the opinion of Dr. Carpenter, that the spermatoza, so called, are not animalcules, but merely cell germs, furnished with a peculiar power of motion. And that they have no more claim to a distinct animal existence, than the epithelia of mucous membrane, which has been seen in movement when separated from the body.—*Elements of Physiology*, p. 154.

Again he writes, "It is clear from the history of their development as well as other considerations, that they cannot justly be regarded in this light, and that they are analagous to the reproductive particles of plants, which, in many cases exhibit a spontaneous movement of extraordinary activity, after they have been set free from the parent structure."—*Op. Cit.*, p. 447.

*In what part of the Generative Apparatus of the Female, do the Spermatoza come in contact with the Ovum to produce Impregnation.*—Dr. Charles Ritchie of Glasgow, believes that the uterus in the human subject, is the normal seat of conception, and that the discovery of spermatoza on the ovaries of quadrupeds, *post coitum*, depends probably on their escape along the tubes, during conception, or on some accidental and rare arrangement of the mucus in their uterine ends, by which it becomes a conducting medium for the passage of the zoospermes.—*Lond. Med. Gaz.—From Am. Jour. Med. Sciences.* Jan. 1846.

Mr. Pouchet advocates a similar theory, and considers the observations of Bischoff and Wagner, who found live spermatoza on the ovaries, to be incorrect. In his own experiments which were chiefly made on rabbits, he found that from the 16th to the 23d hour after copulation, live spermatoza were constantly found in the vagina, but soon after they lost their activity, and by the 25th hour were dead. He was never able to see live spermatoza but a very short distance up the uterine tube, and therefore believes impregnation can only occur in the uterus, or in the (uterine) mouths of the fallopian tubes.—*Rankin's Abstract*, part 1.

Dr. Carpenter is doubtful whether impregnation takes place before the ova leaves the ovarium, or after it has been received into the fallopian tubes, but regards the former as the general rule. It "being quite certain that the spermatoza frequently if not invariably find their way to the ovary." He does not at all refer to the question of uterine conception.—*Physiology*, p. 573.—*Elements of Physiology*, p. 456.

"Bischoff considers it to be immaterial at what part of the fallopian tubes the spermatoza and ovum come in contact, so that they meet before the ovum has reached the uterus, impregnation is sure to take place. He states that he has clearly traced live spermatoza to the ovaries."—*Rankin's Abstract*, part 1.

That fertilization does take place in the ovary and fallopian tubes, is evident from the occurence of extra uterine pregnancies. But nothing on the other hand has been adduced to prove that it does not also occur in the uterus.

